# Control of annual reproductive cycle in the subtropical house sparrow *(Passer domesticus): *evidence for conservation of photoperiodic control mechanisms in birds

**DOI:** 10.1186/1742-9994-3-12

**Published:** 2006-08-22

**Authors:** Amit K Trivedi, Sangeeta Rani, Vinod Kumar

**Affiliations:** 1 Department of Zoology, University of Lucknow, Lucknow 226007, India

## Abstract

**Background:**

In many birds, day length (=photoperiod) regulates reproductive cycle. The photoperiodic environment varies between different seasons and latitudes. As a consequence, species at different latitudes may have evolved separate photoperiodic strategies or modified them as per their adaptive need. We studied this using house sparrow as a model since it is found worldwide and is widely investigated. In particular, we examined whether photoperiodism in house sparrows *(Passer domesticus) *at 27°N, 81°E shared features with those exhibited by its conspecifics at high latitudes.

**Results:**

Initial experiment described in the wild and captive conditions the gonad development and molt (only in captives) cycles over a 12-month period. Both male and female sparrows had similar seasonal cycles, linked with annual variations in day length; this suggested that seasonal reproduction in house sparrows was under the photoperiodic control. However, a slower testis and attenuated follicular growth among captives indicated that other (supplementary) factors are also involved in controlling the reproductive cycle. Next experiment examined if sparrows underwent seasonal variations in their response to stimulatory effects of long day lengths. When birds were transferred every month over a period of 1 year to 16 hours light:8 hours darkness (16L:8D) for 17–26 weeks, there was indeed a time-of-year effect on the growth-regression cycle of gonads. The final experiment investigated response of house sparrows to a variety of light-dark (LD) cycles. In the first set, sparrows were exposed for 31 weeks to photoperiods that were close to what they receive in between the period from sunrise to sunset at this latitude: 9L:15D (close to shortest day length in December), 12L:12D (equinox, in March and September) 15L:9D (close to longest day length in June). They underwent testicular growth and regression and molt in 12L and 15L photoperiods, but not in 9L photoperiod. In the second set, sparrows were exposed for 17 weeks to photoperiods with light periods extending to different duration of the daily photosensitivity rhythm (e.g. 2L:22D, 6L:18D, 10L:14D, 14L:10D, 18L:6D and 22L:2D). Interestingly, a slow and small testicular response occurred under 2L and 10L photoperiods; 6L:18D was non-inductive. On the other hand, 14L, 18L and 22L photoperiods produced testicular growth and subsequent regression response as is typical of a long day photostimulation.

**Conclusion:**

Subtropical house sparrows exhibit photoperiodic responses similar to that is reported for its population living at high latitudes. This may suggest the conservation of the photoperiodic control mechanisms in birds evolved over a long period of time, as a physiological strategy in a temporally changing environment ensuring reproduction at the best suited time of the year.

## Background

Many birds exhibit seasonal cycles in several functions including body mass, plumage color, hormone levels, gonadal growth and development, beak pigmentation, song production, nest building, molt, parasitic load, immune system etc. [1-4]. Most of these functions center on the reproduction, which is timed to occur in the year when the surrounding environment, which itself is seasonal, is maximally favorable for the survival of the offspring [5]. Seasonal activities often appear linked with the changes in environmental photoperiod, although cues like changes in food supply and temperature can influence the timing of a seasonal event [1-7].

The role of day length (=photoperiod) in control of the initiation and termination of events associated with seasonal reproduction is shown in a number of species breeding both at high and low latitudes [for references see [1-9]]. Interestingly, tropical species experiencing relatively smaller amplitude of annual photoperiodic cycle also appear responding to sufficiently small changes in light hours, and they use this cue to time their breeding [3,6,9-11]. Day light interacts with the endogenous daily photosensitive rhythm and regulates growth and development of gonads. Experiments on long day breeding species have established that during spring and summer seasons when daylight exceeds 12 h per day, light later in the day (~12 h after the beginning of the day) coincides with the photoinducible phase of the endogenous circadian (*circa *= about; *dies *= day) rhythm; this causes induction of a photoperiodic response [12]. There is also evidence of the endogenous circannual (*circa *= about; *annum *= day) rhythmicity regulating gonadal cycle in a few species [9,13], although it is unclear yet whether the circannual rhythmicity is completely separate from the circadian rhythmicity. A circannual rhythm may comprise the sequences of mutually exclusive phases, as evidenced from a study on European starlings (*Sturnus vulgaris*) [14]. In starlings held on 12L:12D, circannual rhythms in plasma LH was abolished after castration suggesting that one of the phases of the circannual rhythmicity was dependent on circulating gonadal steroids [14].

The strength of the environmental zeitgeber (*zeit *= time, *geber *= giver; e.g. light-dark [LD] cycle) influences circadian and circannual clocks controlling periodic functions [8]. The photoperiodic environment which acts as zeitgeber for the endogenous clocks underlying seasonality in most bird species [8] varies with the latitudes and seasons. Therefore, a photoperiodic species may exhibit latitude- and season-dependent photoperiodic adaptations. A few studies in fact show this. Great tits *(Parus major) *living in Malaya (3°N) have longer breeding season compared to the north European (>45°N) and British (>51°N) populations [15]. The duration of the postnuptial molt in white-crowned sparrows *(Zonotrichia leucophrys gambelii) *decreases by an average of 2.6 days per degree of increase in latitude (83 days at 35°N compared to 47 days at 49°N; [16]). In a study at 48°N, redpolls brought from 65°N exhibited seasonal response different from its conspecifics that were resident [17]. A study on deer mice *(Peromyscus maniculatus*, [18] suggests that the latitude of origin dictates photoperiodic regulation of the reproductive system. Differences in photoresponsiveness can occur even between populations from similar latitudes, as indicated by studies on blue tits *(Parus caeruleus) *[19,20]. It is likely that such latitudinal or populational differences are purely adaptational, and have not fundamentally changed the physiology of the species. A study of Moore et al. [21] found no difference in GnRH challenged plasma testosterone levels between mid-latitude breeder Puget Sound white-crowned sparrows (*Zonotrichia leucophrys pugetensis) *and high-latitude breeder Gambel's white-crowned sparrows (*Zonotrichia leucophrys gambelii*); interestingly, equatorial rufous-collared sparrow (*Zonotrichia capensis) *exhibited greater plasma testosterone levels than high latitude species.

The fact that both high- and low-latitude species possess sensitivity to even very small changes in photoperiods [1-9] illustrates that inhabiting different photoperiodic environments they necessarily represent adaptations. Studying photoperiodic adaptations in a bird species distributed widely covering different latitudes can therefore be much interesting. The prediction would be that populations living in the north would delay the onset of gonadal growth compared to their conspecifics from the south because of the difference in amplitude of annual photoperiodic cycle the two populations will be exposed to. This may be achieved by (i) the change in the photoperiodic threshold for gonadal growth, and (ii) the adjustment to local cues like temperature and food availability. Silverin et al. [22] tested this in great tits by comparing responses of birds under same lighting conditions from three different latitudes (45°, 57° and 70°N). Birds breeding in the north had higher photoperiodic threshold than those breeding in the south. The results showed greater variability in breeding behavior between the populations of great tits from three different latitudes, and led to conclusion that selection pressure favored photoperiodic cues over non-photoperiodic cues [22]. A study on blue tits supported this by showing that a long photoperiod can override non-photoperiodic factors in timing of the reproduction [23]. Because all three great tit populations employed in the study were still from temperate latitudes (>40°N), a study of the photoperiodic adaptations in a species from subtropics/tropics could be more interesting.

House sparrow *(Passer domesticus) *is found all over the world and is one of most widely investigated bird species. Studies on high latitude house sparrows have established that (i) sparrows are annually breeding species exhibiting seasonal variations in gonadal size and reproductive hormones including levels of GnRH (gonadotropins releasing hormone) and GnIH (gonadotropin inhibitory hormone) [24-26], (ii) day length controls the timing of the initiation and termination of the annual gonadal cycle [27-30], and (iii) photoperiod-induced gonadal function is mediated by the circadian rhythms [31-33]. Further, there could be differences in the breeding seasons among house sparrow populations from relatively similar latitudes. Murton and Westwood [34] in their study on British sparrows found Surrey (51°N) sparrows in more advanced stage of their vernal recrudescence than Cambridgeshire (52°N) sparrows at the same time of the year. The difference in the timing of breeding of sparrows was probably due to the differences in food availability as a consequence of ambient temperatures at two places, and not due to photoperiodic conditions which were almost the same. Experimentally, it has been shown that the duration and timing of food availability influences the rate of photoperiodic induction of testicular growth in house sparrows [35]. Further, a study on four populations of house sparrows [36] spread over a latitudinal range of 18° (between 34°N and 52°N) concluded that the durations of spermatogenesis and photorefractoriness varied with the latitude. As latitudes increased, the duration of spermatogenesis shortened and the duration of photorefractoriness lengthened.

Hence, with large data sets from the populations around the world, house sparrow presents an ideal opportunity to investigate if the photoperiodic strategy involved in regulation of avian seasonal cycles is conserved. That is, whether the fundamental property of photoperiodic cue response system is retained by house sparrow although its populations inhabiting different latitudes could generate appropriate adjustments to local conditions such as weather, food availability, temperature, etc. To test this, we carried out the present study on house sparrow at 27°N, 81°E, since no such detailed study has ever been done at these latitudes. The general prediction was that house sparrows at this latitude (27°N) will show photoperiodic responses more like to those at 34°N, reported above [36]; i.e. they will have longer breeding season as indicated by the growth and regression of gonads under experimental conditions. In different experiments, we have (i) described the annual gonad development cycle in the wild and captive conditions, (ii) examined the changes in responsiveness to a long day length (16 hours light:8 hours darkness; 16L:8D) over different months of the year, and (iii) investigated the induction and regression of gonadal responses under different photoperiods.

## Results

**Figure 1:**
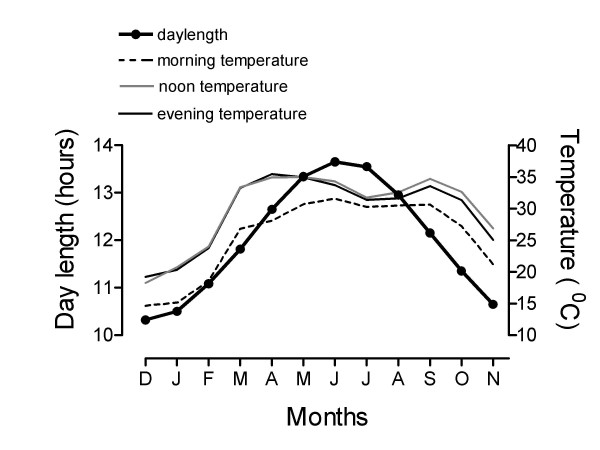
Variations in lighting and temperature conditions at 27°N, 81°E over the 12 month period, from December 2001 to November 2002 recorded in middle of every month. Data on day length are plotted for 15^th ^of each month. Data on temperature were recorded in middle of the aviary at 0800 h, 1200 h and 1700 h.

Figure 1 shows annual variations in the length of daily light period from sunrise to sunset and temperature in the middle of the aviary measured three times in the day: at 0800 h, 1200 h and 1700 h. At 27°N, 81°E, the range of annual variation in day length was about 3.5 hours. Daily temperature cycle also exhibited a seasonal pattern.

### Series A: Gonad development cycle under natural day lengths: effect of captivity

#### Experiment 1

**Figure 2:**
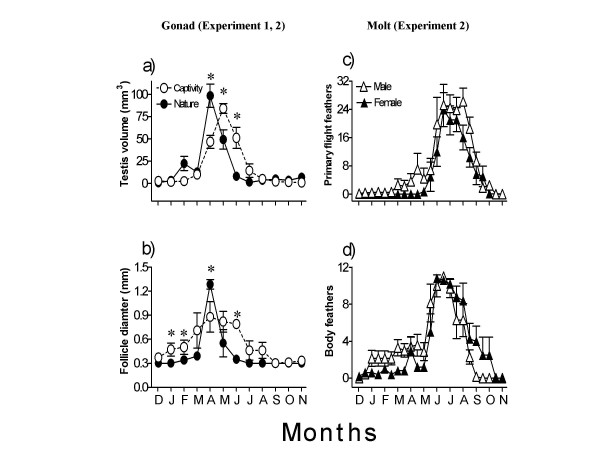
Mean (±SE) testis volume (a), follicle diameter (b), and molt scores of primary flight (c) and body feathers (d) of male and female house sparrows procured every month from the wild or kept in the out door aviary from December through November. In captives, peak testicular growth was delayed by 4 weeks (a), and follicular growth was attenuated (b). Also, the shape of curve reflecting ovarian growth and regression was difference between wild and captive sparrows (b). Asterisks show significant difference (P < 0.05) in the response between the two conditions.

Figure 2 presents data on testis volume and follicle diameter of birds captured each month from the wild. Testes underwent significant annual growth-regression cycle (F_11,102 _= 24.15, P < 0.0001, 1-way ANOVA; Fig. 2a). In December, all birds had regressed testes, but a month later (January) 5 of 10 birds had slightly initiated testicular growth (TV = 3.20 ± 0.80 mm^3^) and 2 months later (February) 9 of 10 birds had moderately grown testes (TV = 22.0 ± 8.00 mm^3^). When captured in March, few birds in the group still had unstimulated testes, which reflected asynchronous testicular growth within the population. All birds captured in April, however, had fully grown testes (TV = 98.4 ± 13.00 mm^3^). Testicular regression had begun by May; half of birds examined in this month had smaller testes (small to moderate response). June birds had variable testicular regression, but July birds had fully regressed testes. In between August and October, half of the sparrows captured had regressed testes and the remaining half had slightly larger testes (TV =< 9.82 mm^3^). In November, the number of birds with regressed testes further declined.

Ovary underwent similar significant annual growth-regression cycle (F_11,88 _= 43.04, P < 0.0001, 1-way ANOVA; Fig. 2b). All sparrows examined from December to February had fully regressed (indistinct) follicles. In March, 3 of 10 sparrows had slightly enlarged follicle, but in April, all sparrows had fully enlarged follicle (FD = 1.29 ± 0.06 mm). By May, majority of sparrows had regressed their ovary (FD = 0.55 ± 0.17 mm); ovary remained regressed in subsequent months of June to November (Fig. 2b).

#### Experiment 2

Sparrows held in captivity underwent similar significant growth-regression cycle of gonads (testis: F_11,44 _= 32.69, P < 0.0001; ovary: F_11,66 _= 5.692, P < 0.0001, 1-way RM ANOVA; cf. Figs. 2a and 2b). Testes were stimulated in March, and grew to full size by May. Regression began by June, and testes were fully regressed by August (Fig. 2a). Similarly, follicular growth was initiated in March, and enlarged follicles were found from April to June. Thereafter, regression began and by September all birds had fully regressed ovary with indistinct follicles.

Molt of wing primaries and body feathers of both sexes progressed with gonadal regression (cf. Figs. 2a–d). When compared over 12 months, molt scores of wing primaries and body feathers showed significant variation (male: F_22,88 _= 14.16, P < 0.0001 [primaries], F_22,88 _= 10.87, P < 0.0001 [body]; female: F_22,132 _= 18.85, P < 0.0001 [primaries], F_22,132 _= 12.47, P < 0.0001 [body]); maximum scores were found in July and August and minimum in November. Individuals differed in the timing of the onset of molt, but all members of the group completed molt by October or November.

A comparison of gonadal growth-regression cycles between two experiments by 2-way ANOVA (factor 1: condition – wild vs. captive; factor 2, season of the growth and regression) revealed a significant effect of the condition (F_11,155 _= 11.88, P = 0.0007) in female but not in male sparrows (F_11,151 _= 0.0250, P = 0.8745). However, the seasons (males: F_11,151 _= 30.60, P < 0.0001; females: F_11,155 _= 20.49, P < 0.0001) and the interaction between the condition and season (males: F_11,151 _= 6.727, P < 0.0001; females: F_11,155 _= 4.427, P < 0.0001) had significant effects in both sexes.

### Series B: Season-dependent variation in sensitivity of the photoperiodic response system

**Figure 3:**
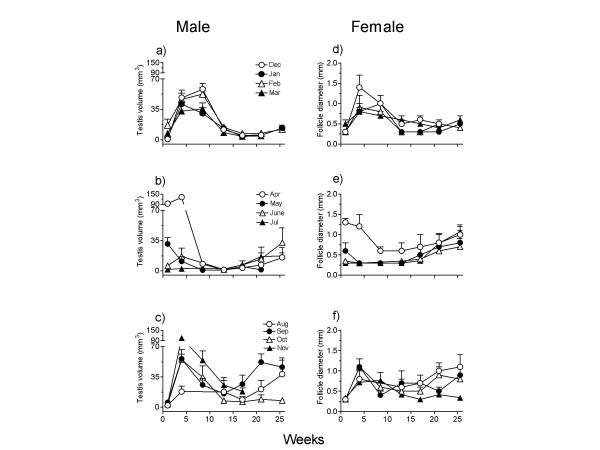
Mean (±SE) testis volume (a-c) and follicle diameter (d-f) of house sparrows (n = 5 – 9 each) procured from the wild every month, and transferred to 16 hours light :8 hours darkness (16L:8D) from December 2002 to November 2003 for a period of 17 to 26 weeks. Data are plotted in groups of 4 months for the sake of clarity. There was a time-of-year effect on the gonadal response to 16L:8D, which suggested that physiological mechanisms underlying reproductive cycle in house sparrows underwent seasonal changes.

Figure 3 shows results. Sparrows underwent significant change in gonadal size (growth or regression depending on the time of the year) when exposed for 17 to 26 weeks to long day lengths (16L:8D) each month over a 12-month period (December: F_5,35 _= 37.83, P < 0.0001; January: F_6,42 _= 21.60, P < 0.0001; February: F_6,24 _= 18.45, P < 0.0001; March: F_5,40 _= 7.98, P < 0.0001, April: F_6,48 _= 48.25, P < 0.0001; May: F_5,20 _= 11.40, P < 0.0001; June: F_5,20 _= 2.795, P < 0.05; July: F_6,24 _= 2.870, P = 0.0298; August: F_5,30 _= 3.053, P = 0.0241; September: F_6,36 _= 6.494, P < 0.0001; October: F_6,24 _= 12.07, P < 0.0001; November: F_4,20 _= 32.21, P < 0.0001; 1-way RM ANOVA). During December through March, testes grew in size in the first 4 – 9 weeks of 16L:8D (Fig. 3a), and then regressed. In April sparrows when they already had recrudesced testes (TV = 93.77 ± 12.10 mm^3^), 16L first induced further growth (TV = 131.7 ± 14.0 mm^3^, P < 0.001, Newman-Keuls test) and then an abrupt regression (Fig. 3b). 16L did not prevent testicular regression in May birds; they became fully regressed by the end of 9 weeks of exposure (Fig. 3b). June and July sparrows were partially recrudesced in 17 weeks (Fig. 3b). August birds showed a small initiation of testis growth (F _5,30 _= 3.053, P = 0.0241; 1-way RM ANOVA) but in birds of September through November, a full testicular growth (P < 0.0001) occurred within 4 weeks; this indicated that by this time photorefractoriness was completely broken (Fig. 3c).

Similar significant changes in follicular diameter occurred in all months of the year, except in June (Figs. 3d–f). (December: F_5,35 _= 6.731, P = 0.0002; January: F_6,30 _= 6.185, P < 0.0001; February: F_6,18 _= 6.087, P = 0.0131; March: F_6,48 _= 2.631, P = 0.0275, April: F_6,36 _= 4.703, P = 0.0013; May: F_6,30 _= 2.989, P = 0.0207; June: F_5,15 _= 1.190, P = 0.3601; July: F_6,36 _= 8.267, P < 0.0001; August: F_5,25 _= 3.178, P = 0.0235; September: F_6,36 _= 5.809, P = 0.0003; October: F_6,48 _= 4.691, P = 0.0008; November: F_6,24 _= 2.642, P < 0.05; 1-way RM ANOVA). Ovarian growth occurred within 4 to 9 weeks of 16L:8D in birds of December through March (Fig. 3d). In April birds with developed ovary, follicles regressed in 9 weeks of 16L (Fig. 3e). Birds subjected to 16L in May through July did not show photoinduction, except a small initiation at the end of the experiment in July birds (Fig. 3e). 16L caused a significant (P < 0.05, Newman Keuls test) increase in follicular diameter within 4 weeks in the month of August through October, which indicated regaining of the photoresponsiveness (Fig. 3f). November group however had variable response; 2 of 5 individuals did not show follicular growth at least until 22 weeks of exposure (Fig. 3f).

### Series C: Response to varying light-dark (LD) cycles

#### Experiment 1

**Figure 4:**
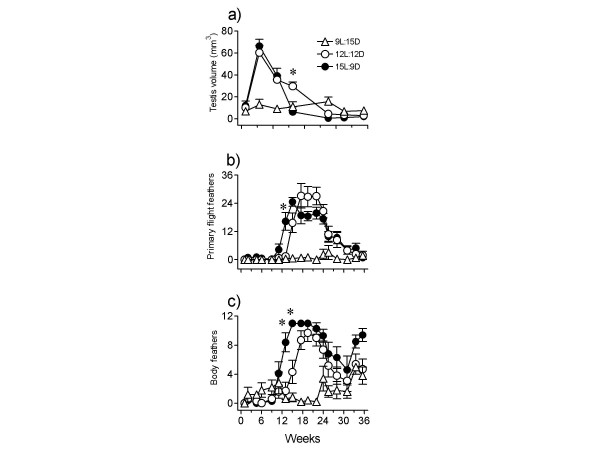
Mean (±SE; n = 7–8) testis volume (a) and molt score of primary flight (b) and body feathers (c) of male house sparrows exposed to a short day length (9L:15D), equinox day length (12L:12D), and long day length (15L:9D) for a period of 36 weeks. Whereas 9L did not induce growth-regression of gonads or molt, 12L and 15L did. Also, photoperiodic induction and hence subsequent regression and molt were earlier in 15L than in 12L. Asterisks show significant difference between groups on the day of observation (P < 0.05)

Figure 4 shows results. Testes did not undergo growth-regression cycle in birds exposed to 9L:15D (F_6,56 _= 0.9426, P = 0.4723; 1-way RM ANOVA). But, a full recrudescence-regression cycle occurred in those exposed to 12L:12D and 15L:9D (12L:12D – F_6,63 _= 38.56, P < 0.0001; 15L:9D – F_6,56 _= 35.31, P < 0.0001; 1-way RM ANOVA). The timing and magnitude of peak testis response were similar in 12L and 15L photoperiods. However, testes regressed relatively slowly under 12L:12D (Fig. 4a); fully regressed testes were found by week 22 of 12L compared to week 13 of 15L.

Data on molt scores of birds under 9L:15D revealed the absence of molt in wing primaries and a partial and slow molt of body feathers (only body feather molt scores were distinct by the end of 24 weeks) (Fig. 4b,c). On the other hand, birds had undergone significant molt under 12L and 15L photoperiods (12L:12D: body – F_14,135 _= 20.82, P < 0.0001; wing primaries – F_14,135 _= 12.11, P < 0.0001; 15L:9D: body – F_14,120 _= 14.85, P < 0.0001; wing primaries – F _14,120 _= 24.05, P < 0.0001; 1-way RM ANOVA). Consistent with data on testes, the onset of molt of wing primaries and body feathers under 15L was significantly earlier than under 12L (P < 0.05; Student's t-test) although molt completed almost at the same time in both the groups (cf. Figs. 4b,c). A comparison by 2-way ANOVA also indicated significant difference between 12L and 15L groups in the molt of body feathers (F_1,255 _= 32.38, P < 0.0001) but not of wing primaries (F_1,255 _= 0.4337, P = 0.5108).

#### Experiment 2

**Figure 5:**
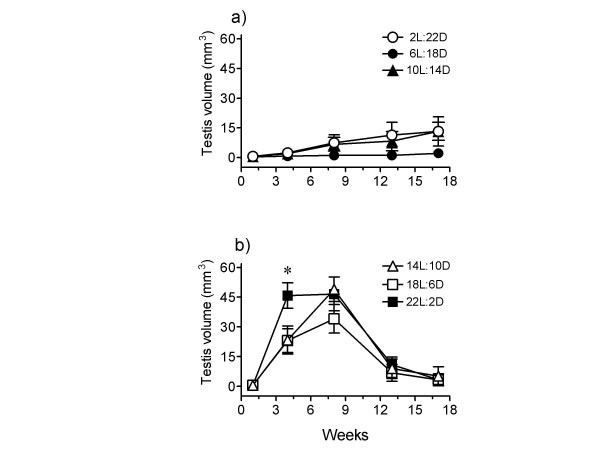
Mean (±SE) testis volume of male house sparrows (n = 9–10) subjected for 17 weeks to photoperiods with increasing lengths of daily light periods. **a: **2L:22D, 6L:18D and 10L:14D. A slow and small testicular response occurred in 2L and 10L, but not in 6L photoperiod, except that 2 of 7 birds in this group showed small initiation at the end of the experiment. **b: **14L:10D, 18L:6D and 22L:2D. A faster induction of testicular growth occurred in 22L, but regression was almost the same in all three photoperiods. Asterisk shows significant difference between 22L and 18L or 14L groups.

Figure 5 shows results. A significant testicular growth-regression cycle occurred during 17 weeks of 14 L, 18L and 22 L photoperiods (14L:10D – F_4,28 _= 13.52, P < 0.0001; 18L:6D – F_4,24 _= 10.02, P < 0.0001; 22L:2D – F_4,24 _= 22.45, P < 0.0001; 1-way RM ANOVA). The magnitude of response among 14L, 18L and 22L groups was similar, but the rate of testicular growth was significantly faster (P < 0.05; 1-way ANOVA) in the 22L group. The timing of testis regression was interestingly the same in all of them (Fig. 5b). Surprisingly, 1-way RM ANOVA of the data from short photoperiods showed significant increase in testis size in 2L and 10L photoperiods, but not in the 6L photoperiod (2L:22D – F _4,24 _= 2.886, P < 0.05; 6L:18D – F_4,24 _= 2.012, P = 0.1249; 10L:14D – F_4,28 _= 3.206, P = 0.0275). The response to 2L:22D and 10L:14D was nonetheless significantly slower (P < 0.05; F-test), smaller and variable (cf. Figs. 5a,b). At the end of the experiment, the response among 2L birds varied from full response (n = 2) to initiation of response (n = 3) to no response (n = 2). Similarly, among 10L birds it varied from full response (n = 1) to moderate response (n = 1) to small response (n = 2) to initiation of response (n = 4). In nonstimulatory 6L:18D, 5 of 7 birds never responded, and of the remaining 2 birds, one had small and the other had slightly initiated response.

## Discussion

There appears a close correspondence between the gonad development cycle of house sparrow and annual variations in day length (cf. Figs. 1 and 2). Increasing photoperiods (≥12 h per day) of spring months (March, April) triggered the gonadal growth, but then gonads regressed during summer months (June or July) when day lengths were still much longer than of spring months. Similar to that described in the annual reproductive cycles of many species including temperate house sparrows [8,27,37], this post-reproductive refractory period suggests that the physiological mechanisms controlling reproductive cycle in the subtropical house sparrows undergo dramatic changes in their response to day length. Interestingly, a study of Misra et al. [38] on blackheaded bunting *(Emberiza melanocephala) *showed the evidence of seasonal changes in photoresponsiveness even when birds were maintained on a non-stimulatory short photoperiod, which is widely used to ensure photosensitivity in a photoperiodic species. In buntings maintained since February on 8L:16D and subjected to 16L:8D from March to August, the magnitude of testicular response declined as short days progressed until July when the response was restored.

Figure 2 shows that the gonad development and molt cycles of house sparrow at 27°N, 81°E closely compare with those of its population living at high latitudes. A study on sparrows at 52°N reported that testes grew in size steadily until May, remained large in June and were fully regressed by late August [27]. In this study, one-fifth of sparrows began molt of their primary flight feathers by late July, all were molting a month later, and the molt was complete by early November. Compared to this, our sparrows had largest gonads in May and were fully regressed in July. Molt began in June, progressed steadily and was almost complete by late September. Thus, sparrows at 27°N appeared exhibiting a photoperiodic strategy in regulation of their reproductive cycle, similar to their high latitude conspecifics (Figs. 2a,b). This is interesting since a previous study on Indian house sparrows at 22°N, 88°E reported a longer breeding season lasting for 10 months [39]. The difference in reproductive cycle of these two Indian studies could be attributed to the difference in the food availability as a consequence of the differences in changes in temperature over seasons at these two latitudes, similar to that reported for high latitude birds [34,35,40,41]. In Threadgold's study also, sparrows of 34°N had longer breeding season than those of 52°N [36]. Whatever explanation is offered, these findings tend to suggest that actual breeding strategy in birds is modified by local conditions of the given latitude.

A comparison of gonad development cycle between the wild and captive sparrows revealed that in captives (i) the attainment of peak testicular growth was delayed, (ii) the amplitude of follicular growth was attenuated, and (iii) overall duration of the gonadal growth phase was longer (Figs. 2a,b). These differences could have occurred due to one or all of the following reasons. (1) The two conditions were different in terms food availability. Whereas wild sparrows had free access to all kinds of food in its environment, captive sparrows had fixed diet of seeds of *Setaria italica *and *Oriza sativa*. Several reports suggest the effects of food availability on the gonadal development [40,40-42]. (2) Absence of supplementary factors including opportunity of pair-formation to captives modified their gonad development cycle [1,43]. (3) Confinement within the aviary (size = 3.0 × 2.5 × 2.5 m^3^) may have been stressful; this in turn affected the activity of hypothalamo-hypophyseal-gonadal axis, and hence the timing and magnitude of gonadal growth and development in captives [44,45].

Results of the series B experiments further support the idea of seasonality in photoresponsiveness of house sparrows (Fig. 3). A 16L photoperiod did not prevent the collapse of large gonads in May birds or re-initiate recrudescence of regressed gonads in June and July birds, but caused recrudescence in September sparrows. This suggested that there was a seasonality in loss and gain of photoresponsiveness in sparrows. A comparable situation exists in several photoperiodic species including temperate zone grey partridge [*Perdix perdix*, [46]] and subtropical Indian weaver bird (*Ploceus philippinus *[47]) and brahminy myna (*Sturnus pagodarum *[48]). A study of Dawson [27] on house sparrows at 52°N, nevertheless, provided slightly different results. In this study, sparrows shifted to 18L:6D in June did not regress their testes at least for the next 25 days; in fact, by mid-July testes under 18L were significantly larger than those in natural day length at this time. The difference between our results and those of Dawson [27] could be due to difference in the amplitude of photoperiodic cycle which sparrows experienced at these two latitudes as well as due to local conditions such as temperature and food availability. It is known that ambient temperature significantly affects the photoperiodic induction of gonadal development and regression in birds [49,50].

Data presented in figure 4a are consistent with the idea that longer the photoperiod, faster is the rate of gonadal growth and subsequent regression; hence, gonadal growth phase becomes narrower [38,51-53]. In the present study, the rate of photoperiodic induction and subsequent regression was faster in 15L than in 12L photoperiod (cf. Fig. 4a). Although the timings of the peak testicular response were not different between 12L and 15L groups (Fig. 4a), probably because a 4-week interval of first observation was long enough to allow full testicular growth under both the photoperiods, the timing of the onset of testicular regression and molt clearly showed a faster photoinduction under 15L:9D (Fig. 4). Testes regressed (cf. data on week 13, Fig. 4a) and molt began earlier in 15L than in 12L photoperiods (Fig. 4b,c). Thus, as one would expect, molt followed the testicular regression: faster was the testicular regression, earlier was the onset of the molt (cf. Fig. 4a–c). A similar molt pattern has been reported in house sparrows of 52°N subjected to stimulatory photoperiods [27]. Photoperiodic control of molt is also reported in the European starling [54]. A close relationship between the gonadal regression and post-nuptial molt is known in several species [55]. The present results are also comparable to those reported on house sparrows at 52°N exposed to 18L, 16L and 13L photoperiods [28]. In this study, as compared to that in 16L and 18L, peak testis growth in 13L was delayed by 3 weeks. Similarly, testes regressed by 9, 12, 18 weeks in 18L, 16L and 13 L groups, respectively [28]. Figure 5, however, shows that in sparrows exposed to 22L, 18L and 14L, testes recrudesced faster in the 22L photoperiod, as one would expect, but the subsequent regression was not different between the three photoperiods. It appears that the duration of testicular growth phase under stimulatory photoperiods is not altered when daily light period far exceeds the photoperiodic threshold (cf. Fig. 4a, 5). In other words, the extension of the light period into the photoinducible phase of the circadian rhythmicity underlying photoperiodism longer than ~2 h each day does not change the gonadal growth phase in house sparrows. This may be logical given the fact that sparrows at 27°N experience a maximum of about 14 h light per day (sunrise to sunset, Fig. 1), and hence the photoinducible phase of circadian rhythmicity in this species [56] is daily exposed to only slight greater than 2 h light period. A study of Rani and Kumar [57] on redheaded bunting *(Emberiza bruniceps) *provided the evidence that illumination of photoinducible phase beyond a critical duration did not enhance the testicular response.

At this latitude (27°N, 81°E), house sparrows closely share habitat with weaver birds. Although there is a difference of about 4 weeks in peak testicular development between the two species (our unpublished data), both of them initiate their gonad development cycle with increasing day lengths of spring. Hence, they are expected to share similar photoperiodic mechanisms, although sparrow may have slightly lower photoperiodic threshold. Indeed, Indian weaver bird at 25°N is reported exhibiting testicular response to 9L, 12L and 15L photoperiods [47,58,59] similar to that is shown by sparrows in the current study (Fig. 4).

On exposure to short photoperiods (≤10 h per day), a long day breeder usually will not show gonadal recrudescence, which is indicative of the importance of photoperiodic cues over endogenous seasonal rhythm in the control of reproductive cycle of a species. However, if a long day breeder exhibits gonadal recrudescence under such short photoperiods, its response may be considered as a consequence of the seasonal rhythm rather than to the photoperiod. Results of the experiment 2 of series III (Fig. 5a) should be viewed against this background. Sparrows exhibited a testicular response, albeit slow and small, to 2L:22D and 10L:14D; however, no response to 6L:18D (cf. Fig. 5a). An earlier study has also reported testicular recrudescence under 1L:23D temperate house sparrows [36]. In the current study, the small induction under 2L and 10L photoperiods could be occurring due to different reasons. A 10 h square-wave of light at bright intensity in 10L:14D regime could be compared to a lighting situation in the natural environment closer to when sparrows would initiate testicular recrudescence. Therefore, sparrows continuously exposed to 10 h light per day exhibited small initiation of response. This is supported by the result under 6L:18D in which no significant enlargement of testes occurred. Several studies have shown spontaneous full growth and regression cycle of gonads under 12 h photoperiods [7]. On the other hand, we interpret small response under 2L:22D as the consequence of the annual rhythm of the growth-regression-regrowth of testes rather than of the photoperiodic condition. It is possible that a short photoperiod like 2L:22D is unable to sustain the entrainment of the endogenous clocks underlying seasonality in house sparrows. The photoperiodic entrainment of the circadian rhythmicity, which can be different under different zeitgeber conditions, sets the timing of the photoinductible phase [60], and the interaction of the latter with the light regulates gonad development cycle in photoperiodic birds [12,31-33].

In general, the timing and amplitude of the photoperiodic response differed between male and female sparrows (cf. Figs. 2a,b). Long days induced full testicular growth but only partial ovarian growth. It appears that increasing photoperiods induce initial slow growth phase and not the final fast or exponential growth phase of ovary, which is influenced by supplementary factors [1,43]; full reproductive competence in females is often determined by the stimuli from mate, nest site and food availability [8,61-63]. Such a difference in photoperiodic regulation of seasonal cycles between sexes probably reflects an adaptive strategy since it restricts ovulation, and hence fertilization, until the time when the chances are favorable for the survival of the offspring.

## Conclusion

House sparrows at 27°N, 81°E show photoperiodic responses similar to those of its populations living at high latitudes, e.g. 52°N. This means that at relatively low-latitudes, house sparrow continues using photoperiodic cues from the environment to regulate their reproductive cycles. Studies on house sparrow show conservation of photoperiodic control mechanisms evolved over a long period of time, as an adaptive strategy in the temporal environment that ensures reproduction at the most suited time of the year. Differences in photoperiodic responses among populations of the same species inhabiting different latitudes could suggest specific adaptations required by a species at the given latitude.

## Methods

Three series of experiments were performed on adult house sparrow *(Passer domesticus) *captured from the wild and kept in an outdoor aviary until the beginning of the experiment. Our outdoor aviaries are situated on the roof of the first floor (at the height of about 3 m from the ground) and receive unrestricted natural lighting and temperature conditions.

### Series A: Gonad development cycle under natural day lengths: effect of captivity

We studied changes in the sizes of testis and ovarian follicle over a 12-month period to describe gonad development cycle in the wild and captive house sparrows in relation to annual variations in day length. If factors other than day length also contributed to mechanisms controlling growth and regression of gonads, then there would be a difference in the magnitude of growth and/or temporal phasing of gonadal cycle between wild and captive sparrows. Two experiments were performed.

#### Experiment 1

Beginning from December 2001 over the next 12 months, the size of testis and ovarian follicle were measured in a group of male (n = 6–10 each) and female (n = 4–10 each) sparrows captured in the middle of every month from the wild.

#### Experiment 2

In December 2002, a group of male and female sparrows (n = 8 each) were kept in the outdoor aviary (size = 3.0 × 2.5 × 2.5 m^3^) for 12 months; at a given time they were in company of another 25–30 individuals that were not the part of the experiment. In aviary, they received unrestricted natural lighting and temperature conditions, although these were not the same, as one would find underneath the bare sky as the aviary was roofed with tin sheets placed about 0.5 meter above the ceiling of the aviary. Birds received natural lights from the three sides (east, north and south). Observations were made fortnightly on body and primary flight feathers, and monthly on the size of testis and ovarian follicle. Three males and one female died before the end of the experiment, and data from them were excluded from the analysis and presentation in the figures.

### Series B: Season-dependent variation in sensitivity of the photoperiodic response system

We investigated whether house sparrow exhibited season-dependent variation in its response to stimulatory long photoperiods. If yes, this might be taken as a physiological reason for the onset and end of growth-regression-regrowth phases of the annual gonadal cycle. In the middle of every month from December 2001 to November 2002, we subjected a group of male and female sparrows (n = 5–9 each) to a long photoperiod (16 hours light:8 hours darkness; 16L:8D) for 17 to 26 weeks. Observations were made on testicular and follicular size at the beginning and the end of the experiment, and at monthly intervals during the experiment.

### Series C: Response to varying light-dark (LD) cycles

This experiment investigated response of male sparrows to day light periods ranging from 2 h to 22 h per day, since the species is distributed worldwide. Beginning in third week of January 2004, two experiments were performed employing wild caught adult males that were acclimatized for 4 days in the outdoor aviary before being subjected to an LD cycle.

#### Experiment 1

Three groups of sparrows (n = 9–10 each) were exposed to photoperiods that were close to what they receive during different phase of the annual cycle at this latitude: 9L:15D (close to shortest day length in December), 12L:12D (equinox, in March and September) 15L:9D (close to longest day length in June). The experiment ran for 31 weeks. Observations were made at the beginning and the end of the experiment, and at appropriate intervals (molt: 2 week; testis size – 4 week) during the experiment.

#### Experiment 2

Six groups of sparrows (n= 7–8 each) were exposed for 17 weeks to photoperiods that contained systematically varying light phase such that it extends to different phases of the daily photosensitivity rhythm (e.g. 2L:22D, 6L:18D, 10L:14D, 14L:10D, 18L:6D and 22L:2D). Observations on testis size were made at the beginning and the end of the experiment, and at intervals of about 4 weeks during the experiment.

Food (seeds of *Setaria italica *and *Oriza sativa*) and water were available *ad libitum*. Male and female birds were always kept separately. In an LD cycle, birds were held in groups of 3 or 4 individuals per cage (size – 45 × 25 × 25 cm) within light-tight boxes (size – 138 × 60 × 56 cm) providing white light produced by fluorescent (Philips) tubes at ~500 lux. Light intensity measurements reflect light illumination at perch level within the cage. Automatic time switches controlled times of light on and light off. Temperature was not strictly regulated, but our photoperiodic boxes are well aerated through inlets and outlets connected to air-circulators, and so temperature inside them does not vary more than 1–2°C from the room temperature.

The size of gonads size was recorded by laparotomy as described in our earlier publications [38]. Briefly, a small incision was made between the last two ribs on the left flank, gonads were located within the abdominal cavity with the help of a spatula, and the length and width of the left testis or the diameter of largest ovarian follicle was measured. Testis volume (TV) was calculated using the formula 4/3π*ab*^*2*^, where *a *and *b *denote half of the long (length) and short (width) axes, respectively. To explain better the induction of a response, we subjectively graded testis size, as described in Kumar et al. [64]: TV = 0.33 to <2.35 mm^3 ^– no response; 2.35 to <9.82 mm^3 ^– initiation of response; 9.82 to <18.86 mm^3 ^– small response; 18.86 to <41.9 mm^3 ^– moderate response; 41.9 mm^3 ^and above – full response. Similarly, a regressed ovary with an indistinct follicle was considered having follicular diameter (FD) of 0.3 mm in order to make data statistically comparable with a stimulated follicle. Molt was studied by scores of feathers of primary flight (wing primaries) and body feathers. As outlined by Boswell [65], we scored primaries in a score of 0–5: 0 – worn or old feather, 1 – missing feather (just dropped), 2 – from a new feather papilla emerging up to attainment of one-third growth, 3 – new feather that has attained two-third growth, 4 – new feather grown, but still growth is incomplete, 5 – new feather fully grown. Thus, each primary could have a maximum score of 5. Because there are nine primaries on each wing, the maximum score for one wing can be up to 45 (9 × 5 = 45), and for each bird the feather score could total up to 90 (2 × 45 = 90). Similarly, minimum score could be as low as 0. For recording body molt, we divided the whole bird's body into 11 different regions: 1 – head, 2 – neck, 3 – shoulder, 4 – back, 5 – pelvic, 6 – throat, 7 – chest, 8 – abdomen, 9 – flank, 10 – shank, and 11 – sub-caudal. Any region could have a score of either 0 (old feathers) or 1 (new feathers emerged), and hence the total body molt score could be in the range of 0–11, depending on the number of regions had scores of 0 or 1.

The data are presented as mean and SEs. They were analyzed using one-way analysis of variance (1-way RM ANOVA) with repeated measures, as appropriate, followed by the post hoc Newman-Keuls test, if ANOVA indicated a significance of difference. Two-way ANOVA was used to compare when two factors (e.g. photoperiod and duration) were involved. Two and three groups at one time point were compared using the student's t-test and 1-way ANOVA, respectively. Significance was taken at P < 0.05.
